# A Case of Cholera, with Remarks

**Published:** 1849-05

**Authors:** W. D. Barnett

**Affiliations:** Camden, Ark.


ART. III.—*A Case of Cholera, with Remarks.* By W. D. BARNETT, M.D. of Camden, Ark.

Camden is a flourishing town built about seven years since and contains a population of between 1000 and 1500. It is situated on the west bank of the Ouachita river, at the head of navigation. It affords excellent navigation and there is a direct and constant communication between this port and New Orleans. The town begins at the bank of the river, where steamboats are landed, and gradually ascends rising over a large hill in a westward direction. The country in this direction is high, dry and well watered by springs. Upon the eastern or opposite side of the river, for a number of miles, the country is low, marshy and interspersed with ponds.

This town, like Natchez, is divided into two parts; one lying upon the hill, the other at its base. It was under the hill, as it is termed, that the case of cholera occurred. The patient applied to me, on the morning of Feb. 7th, 1849, for advice concerning a diarrhea which he said came on him that morning, between midnight and day. The first evacuation, he stated, was very large, and caused considerable exhaustion. After this he went to bed, having but one operation from this time until sunrise, when he arose somewhat debilitated, ate a light breakfast and started to his work, but did not continue at it long before the diarrhea and vomiting returned with greater severity. His pulse was weak, surface cool, no pain on pressure over the region of the bowels, the discharges whitish.

℞ Sub-muriate of mercury grs. 10, opium gr. 1, every two hours, until the vomiting and purging cease. Go home and keep in bed.

At 3 o'clock P. M. I was called to see him and found present every symptom of collapse. The extremities were cold, skin corrugated and livid, pulse imperceptible at the wrist, expired air cool, frequent vomiting and purging of rice water, frequent cramps of the lower and occasionally of the upper extremities.

I gave him calomel 20 grs., opium 1 gr. and camphor 3 grs. every hour; applied sinapisms to the extremities, over the stomach and bowels, and along the course of the spine. Between the doses of the above medicine take brandy with sulph. ether.

7 o'clock P. M. Patient has not vomited since 3 o'clock. All the other symptoms are present and aggravated. He was now ordered 40 grs. of calomel at one dose; discontinue the prescription of cal. opium and camphor. Take a mixture composed of aromatic spirits of ammonia, ether and camphor every hour; practice frictions on the extremities with brandy and mustard; then reapply the sinapisms which before had produced no effect.

10 o'clock P. M. Patient no better; every symptom continues; he has taken 140 grs. of calomel and stimulants in great quantities. He was now bled; about 4 oz. of dark, thick blood was drawn off. Boiling water, after the plan of MacIntosh, was poured upon the extremities; a vesicating plaster was placed along the entire course of the spine from the occiput to the sacrum, and also upon the extremities; continue brandy, ether, ammonia, and camphor every hour.

8th—8 o'clock A. M. Patient still pulseless at the wrist, has, in a great degree lost his consciousness; rice water discharges continue—in a word, all the alarming symptoms.

Withdraw the stimulants in use, and give spirits turpentine 20 drops in camphor water every hour, as long as he can swallow; apply warmth by means of heated bricks to the extremities, and continue frictions.

12 O'clock. Patient shows signs of reaction, the last operation from the bowels being a little discolored with bile; the pulse can be felt at the wrist, heat of the extremities returning; hands and feet blistered from the effects of hot water last night; blister on the spine has drawn well.

3 O'clock. Decided improvement; the last alvine discharge showed that the biliary secretion had returned; a copious secretion of urine has taken place; lividity of the face and extremities vanishing; converses cheerfully and his countenance is becoming natural.

9th. Patient considered convalescent; the alvine dejections through the night continued thick and dark; appetite good. He continued to improve for several days and was able to leave his bed, when, the weather growing suddenly cold, and the poor man living in an open house, pneumonia supervened, and he died a week after he had been pronounced convalescent from cholera.

The question was started here when this patient began to recover, whether his was a case of cholera. I think there can be no doubt that it was. Other cases, similar in all their features and fatal in their results, had occurred in the neighborhood. The symptoms will at once be recognised as unequivocally those of cholera. It is worthy of remark that all the cases here appeared near the steamboat landing. Would they have appeared if there had been no communication with New Orleans? Upon this subject professional opinion is greatly divided, and I have no hope that I could harmonize the views of the different parties by any facts I might bring forward.

March 1st, 1849.

